# Disadvantages of a weight estimation formula for macrosomic fetuses: the Hart formula from a clinical perspective

**DOI:** 10.1007/s00404-018-4917-z

**Published:** 2018-10-04

**Authors:** Christoph Weiss, Peter Oppelt, Richard Bernhard Mayer

**Affiliations:** Department of Gynecology, Obstetrics, and Gynecological Endocrinology, Kepler University Hospital, Johannes Kepler University, Altenberger Straße 69, 4040 Linz, Austria

**Keywords:** Macrosomia, Sonography, Fetal weight estimation, Formula, Hart, Hadlock

## Abstract

**Purpose:**

Sonographic fetal weight (FW) estimation to detect macrosomic fetuses is an essential part of everyday routine work in obstetrics departments. Most of the commonly used weight estimation formulas underestimate FW when the actual birth weight (BW) exceeds 4000 g. One of the best-established weight estimation formulas is the Hadlock formula. In an effort to improve the detection rates of macrosomic infants, Hart et al. published a specially designed formula including maternal weight at booking. The usefulness of the Hart formula was tested.

**Methods:**

Retrospective study of 3304 singleton pregnancies, birth weight ≥ 3500 g. The accuracy of the Hadlock and Hart formula were tested. A subgroup analysis examined the influence of the maternal weight. The Chi-squared test and one-way analysis of variation were carried out. For all analyses, *p* < 0.05 was considered statistically significant.

**Results:**

The overall percentages of births falling within ± 5% and ± 10% of the BW using the Hadlock formula were 27% and 53%, respectively. Using the Hart formula, 24% and 54% were identified within these levels. With the Hart formula, 94% of all weight estimations fall within 4200 g ± 5% and nearly 100% fall within 4200 g ± 10%.

**Conclusions:**

Applying the Hart formula results in an overestimation of fetal weight in neonates with a birth weight < 4000 g and fails to identify high-risk fetuses. We, therefore, do not consider Hart’s formula to be of clinical relevance.

## Introduction

In cases of suspected fetal macrosomia, the physician is always faced with a number of problems in peripartal and labor management, as well as in providing suitable counseling for the expectant parents. Fetal macrosomia is associated with an increased risk of shoulder dystocia, brachial plexus injuries, fractures, and asphyxia of the neonate. The women giving birth are also at-risk of vaginal trauma, anal sphincter rupture, emergency cesarean sections, and postpartum hemorrhagia [1–3]. With average-sized fetuses, the risk of shoulder dystocia is approximately 0.2%. The risk increases to 5% or up to 30% when the birth weight (BW) is in the range of 4000–4500 g or ≥ 5000 g, respectively [4–6].

The literature on macrosomic infants is extensive, with contradictory findings. There is not even a consensus on the definition of macrosomia itself. The prevalence of macrosomia reported in the literature varies from as low as 0.5% up to 15% [7]. In general, macrosomia is defined as a BW ≥ 4000 g. These inconsistencies make it difficult to comprehensively compare the various statements and recommendations that have been made on the topic [8–10].

Large retrospective analyses have not identified any significant differences in adverse birth outcomes between BWs in the range of 3500–4500 g [7, 11, 12]. Only a BW ≥ 4500 g has been associated with an increased risk for perinatal mortality and morbidity. Detecting these fetuses must therefore be of special clinical interest.

A very large number of weight estimation formulas have been published in efforts to detect these at-risk fetuses. Most of the weight estimation formulas that are commonly used underestimate fetal weight when the actual weight exceeds 4000 g [13]. One of the best-established weight estimation formulas used in clinical practice is the Hadlock formula, first published in 1985 [14]. In 2010, Hart et al. [15]. published a formula including fetal measurements such as head circumference, abdominal circumference, femur length, and maternal weight at booking and claimed that it improved detection rates for macrosomic infants. At first glance, the new formula appeared to improve accuracy in comparison with other commonly used weight estimation formulas [16, 17].

The aim of the present study was to investigate and question the usefulness of the Hart formula in clinical practice in a large obstetric department.

## Methods

### Data collection

This retrospective cohort study included all births between January 1, 2013 and December 31, 2015 at the Department of Gynecology, Obstetrics, and Gynecological Endocrinology at Kepler University, Linz, Austria—the largest obstetrics ward in Austria, with around 3800 births per year. The data were obtained from the computer database of perinatal records. The inclusion criteria were: singleton pregnancy, liveborn infants with a BW ≥ 3500 g, a complete dataset of ultrasound examinations, and complete maternal parameters. The most recent estimated fetal weight (EFW) was taken into account. The data represent an unselected cross-section of the population.

Ultrasound examinations form part of routine prenatal management in the department. The examinations were performed transabdominally by experienced physicians, using high-quality ultrasound systems (Voluson E6 and E8, GE Medical Systems, Zipf, Austria).

Women with a normal pregnancy history visit the department for the first time at around 37 + 0 weeks of gestation. A final weight estimation is performed at term. At-risk pregnancies were examined more often, mostly starting in an earlier gestational week, depending on the individual risk profile. In the department, 40 gestational weeks plus 10 days is the longest time period accepted for postdate gestation, after which induction of labor is recommended.

Routine weight estimation included measurements of the biparietal diameter, head circumference, abdominal circumference (AC), and femur length in accordance with the International Society of Ultrasound in Obstetrics and Gynecology (ISOUG) recommendations [18]. The Hadlock formula [14] is routinely used for weight estimation.

Maternal weight at booking and height were obtained from entries in the pregnancy pass issued by the gynecologist at the booking visit. Weight at term was measured routinely in the department.

For this study, the EFW measured using the Hadlock formula was compared with the EFW measured using the Hart formula by completing the same fetal measurements and the maternal weight filed at booking.

Gestational age was assessed relative to the crown–rump length (CRL) measured at the first-trimester ultrasound examination. If the CRL was not known, the first day of the last menstrual period was used for gestational age assessment.

Delivery data such as BW, mode of delivery, Apgar scores, and venous and arterial umbilical cord pH were filed within 1-h after delivery by midwives, in a separate database. Missing data were obtained from a manual review of clinical reports.

### Ethical approval

According to the guidelines and affirmed by a written statement by the chairman of the Research Ethics Committee of Upper Austria, no specific ethical approval was necessary for this retrospective study.

### Statistics

The accuracy of the two formulas for predicting fetal macrosomia (defined as BW ≥ 4000 g) was tested using sensitivity, specificity, false-positive rate (FPR), false-negative rate (FNR); and percentage error (PE): (EFW−BW/BW × 100), absolute percentage error (APE): (|EFW−BW|/BW × 100), positive predictive value (PPV), negative predictive value (NPV), positive likelihood ratio (LR+) defined as sensitivity/(1-specificity), negative likelihood ratio (LR−) defined as (1-sensitivity)/specificity, and overall accuracy defined as (true-positive plus true-negative)/all cases.

Percentages of EFWs falling within the ± 5% and ± 10% levels of the actual BW were calculated for both formulas.

A subgroup analysis examined the influence of the maternal body mass index (BMI) on the detection rate of macrosomic infants using the Hart formula. Three subgroups were compared (BMI < 18.5, *n* = 147; BMI 18.5 ≥ 30, *n* = 2702; and BMI > 30, *n* = 455).

Statistical analysis was carried out using the R statistical software package [19]. The Chi-squared test and one-way analysis of variation (ANOVA) were carried out. For all analyses, *p* < 0.05 was considered statistically significant.

## Results

A total of 3304 births met the inclusion criteria; 779 infants (23%) had a BW ≥ 4000 g, 69 (2%) ≥ 4500 g, and only three infants weighed more than 5000 g.

Among the macrosomic infants (BW ≥ 4000 g), 182 (23%) were identified using the Hadlock formula, with a false-positive rate of 4%. In contrast, 765 (98%) were detected using the Hart formula, with a false-positive rate of 92%. Three of the infants weighing more than 4500 g were correctly detected with the Hadlock formula. The time intervals between measurement and birth in these three cases were less than 1 day, 1 day, and 3 days. Only one infant weighing more than 4500 g was detected with the Hart formula, at an assessment 3 days before labor. None of the infants ≥ 5000 g was detected by either formula. The median time interval between measurement and birth in the infants ≥ 4500 g was 8 days (± SD 7.28 days). In general, the median interval between ultrasound examination and birth was 9 days (± SD 7.11 days).

Gestational diabetes was present in 16.5% of the cases; 7.5% of the mothers did not undergo an oral glucose tolerance test (OGTT) as recommended. Only 15 mothers had preexisting diabetes. Table 1 lists the demographic and obstetric characteristics.

**Table 1:** Demographic and clinical parameters in the study population (*n* = 3304), given as mean (± SD); parity is given as median (range)

Maternal age (years)	29.99 (± 5.22)
Body mass index at booking (kg/m^2^)	24.65 (± 5.16)
Weight at booking (kg)	68.89 (± 15.06)
Weight change during pregnancy (kg)	15.07 (± 6.14)
Parity	2 (1–9)
Gestational age at delivery (weeks)	40.04 (± 2.54)
Time from fetal weight estimation to delivery (days)	9.55 (± 7.11)
Birth weight (g)	3829.34 (± 262.40)
Gender (male/female)	1872/1432

Using the AC as a single predictor for macrosomia, with a cut-off value of 351 mm as suggested by Hart et al. led to a sensitivity of 65% and a specificity of 66% (PPV 37%, NPV 86%). Hart et al. reported sensitivity and specificity levels of 75% and 73% for this criterion (PPV 32%, NPV 94%). Because of these very moderate values, EFW data with an AC less than 351 mm were not excluded. The individual classification parameters for each formula were determined with and without the AC cut-off value as an inclusion criterion. The parameters are shown in Table 2.

**Table 2:** Classification parameters for macrosomia in each formula, with and without the abdominal circumference cut-off value (351 mm) proposed by Hart et al

	Hadlock	Hart
Without AC cut-off		
Sensitivity	23.36%	98.20%
Specificity	95.96%	8.04%
PPV	64.08%	24.78%
NPV	80.23%	93.55%
FNR	76.64%	1.80%
FPR	4.04%	91.9 6%
Overall accuracy	78.84%	29.30%
LR+	5.78	1.07
LR−	0.80	0.22
With AC cut-off		
Sensitivity	35.83%	99.80%
Specificity	88.26%	1.50%
PPV	64.08%	37.20%
NPV	70.17%	92.86%
FNR	64.17%	0.20%
FPR	11.74%	98.50%
Overall accuracy	68.92%	37.76%
LR+	3.05	1.01
LR−	0.73	0.13

*AC* abdominal circumference, *FNR* false-negative rate, *FPR* false-positive rate, *LR *−  negative likelihood ratio, *LR *+  positive likelihood ratio, *NPV* negative predictive value, *PPV* positive predictive value

Table 3 shows the mean PE and mean APE values.

**Table 3:** Mean percentage error and mean absolute percentage error values with the Hadlock and Hart formulas for all births, classified into fetuses with birth weights < 4000 g and ≥ 4000 g

	All births (*n* = 3304)		BW < 4000 g (*n* = 2525)		BW ≥ 4000 g (*n* = 779)	
	Mean PE (± 2 SD)	Mean APE (min; max)	Mean PE (± 2 SD)	Mean APE (min; max)	Mean PE (± 2 SD)	Mean APE (min; max)
Hadlock	− 8.80 (± 8.98)	10.34 (0.03; 2.85)	− 8.00 (± 8.66)	9.66 (0.03; 47.42)	− 11.39 (± 9.50)	12.42 (11.04; 30.60)
Hart	+ 8.55 (± 6.62)	9.45 (0.02; 5.07)	+ 11.28 (± 5.94)	11.28 (6.11; 11.02)	− 0.31 (± 6.62)	3.49 (1.20; 14.32)

*APE* absolute percentage error, *BW* birth weight, *PE* percentage error

**Fig. 1:**
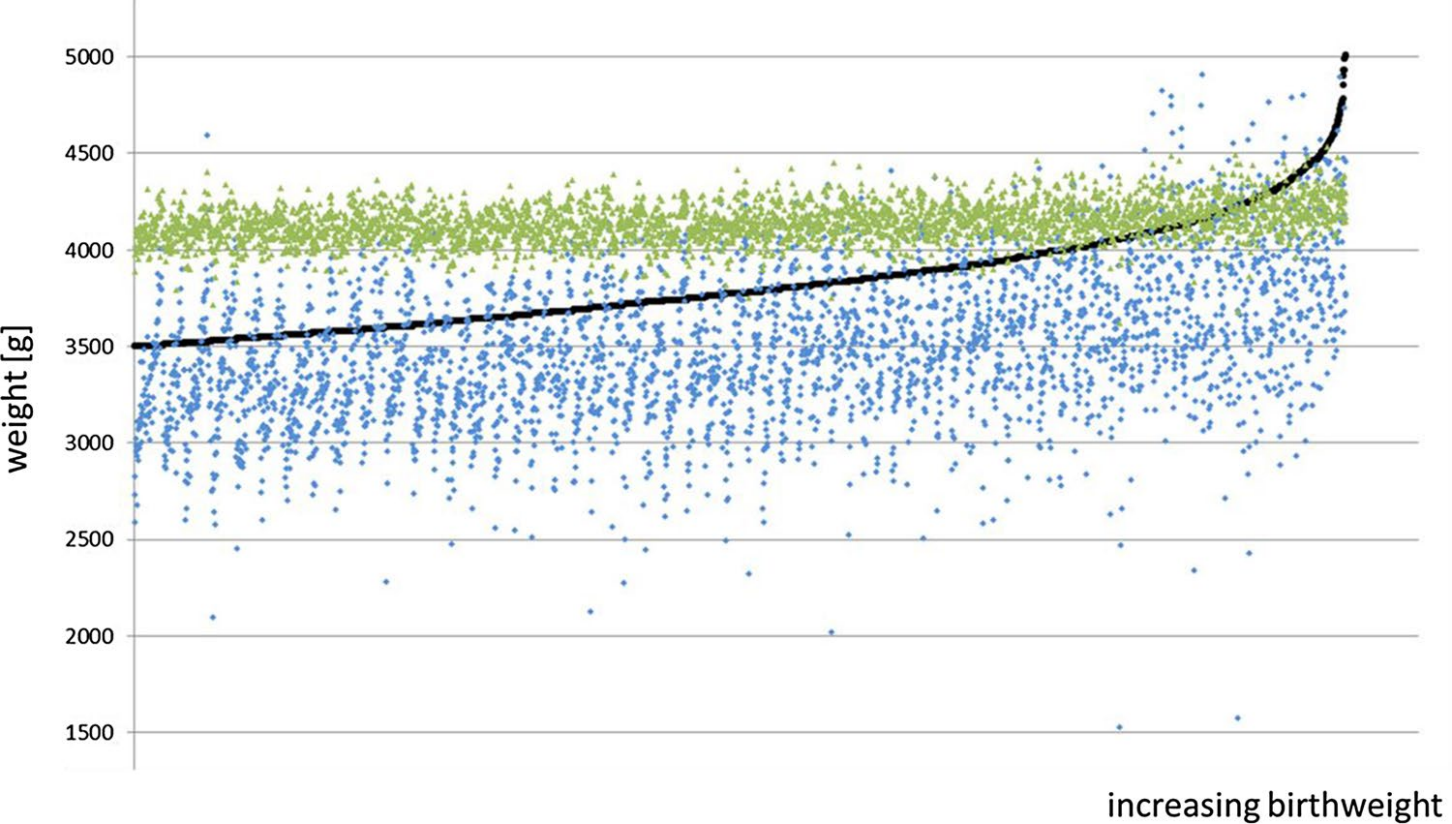
Dot plot of all births (*n* = 3304), ranked by increasing birth weight (BW). In all, 182 (23%) of the macrosomic infants (BW ≥ 4000 g) were correctly identified using the Hadlock formula; 765 (98%) were detected using the Hart formula. The favorable appearance is achieved by the fact that the Hart formula sets the estimated weights within a narrow range around 4200 g. Ninety-four percent of all weight estimations using Hart fall within 4200 g ± 5%, leading to massive overestimation of fetal weight (FW) in children under 4000 g. In addition, both formulas fail to detect high-risk fetuses with a BW > 4500 g. Black dots: actual BW; green dots: FW estimated with the Hart formula; blue dots: FW estimated with the Hadlock formula

The overall percentages of births falling within ± 5% and ± 10% of the BW using the Hadlock formula were 27% and 53%, respectively; the corresponding figures for the Hart formula were 24% and 54%. For infants with a BW < 4000 g, 29% of the Hadlock estimates were within ± 5% of the BW. Only 8% of the Hart estimates were in that range. For macrosomic infants, 20% of the Hadlock estimates and 76% of the Hart estimates were within ± 5% of the BW; 42% (Hadlock) and 98% (Hart) were within ± 10%. In contrast, 94% of all weight estimations using the Hart formula were within 4200 g ± 5% and nearly 100% were within 4200 g ± 10% (Fig. 1).

The influence of maternal BMI on the PE with the Hart formula was not significant (*p* = 0.38).

## Discussion

Large numbers of fetal weight estimation formulas have been published in recent decades—but new formulas are still being added every year [20]. Virtually every measurable fetal or maternal factor has been incorporated into such formulas. They include two-dimensional and three-dimensional formulas, and even formulas without any fetal ultrasound measurements at all [21]. The aim has always been to provide obstetricians with a better tool for fetal weight estimation, allowing the best possible support to be provided for the expectant parents. However, the sheer number of formulas published so far shows that an ideal formula may not actually exist. Not everything that appears to be significant in a published report proves to be of clinical relevance.

Almost every newly developed weight estimation formula has been compared with one of the formulas developed by Hadlock in the 1980s. When one considers the technological circumstances in which Hadlock had to develop his formulas, it seems astonishing that none of the more recent formulas has been able to replace them in obstetric practice. Only recently, Aviram et al. [22] showed that Hadlock’s formula ranked highest for detecting large-for-gestational age neonates in comparison with 20 other formulas. In addition to Hadlock, there are of course other widely used estimation formulas—e.g., the formulas by Shepard [23] and Merz [24], published in 1982 and 1988.

Despite the poor performance of weight estimation formulas in macrosomic fetuses in general [13, 25], estimating fetal weight is an essential part of everyday routine work in an obstetric department in the effort to detect infants who are at risk. Suspected macrosomia always alters the approach to a pregnancy in relation to possible perinatal risks and unwanted effects [26].

It has been shown that labor abnormalities such as arrested labor are more likely to be diagnosed and that cesarean delivery (CD) rates are higher when fetal macrosomia is suspected, despite the real BW [8, 27–29]. Little et al. [30]. showed that the simple fact of performing an estimation within 1 month before delivery is an independent risk factor for CD, and that the CD rate increases by up to 2.5 times when the EFW is ≥ 4000 g, regardless of the actual BW [31]. This is of particular concern, since it is a matter of controversy whether inducing labor or carrying out a CD merely because of suspected macrosomia is beneficial for the mother and child [32]. Several authors have concluded that suspected macrosomia alone does not justify induction of labor or primary CD [12, 33, 34]. In addition, maternal complications increase without benefit for the infant [26]. According to the American College of Obstetrician and Gynecologists (ACOG) guidelines, suspected fetal macrosomia is not an indication for inducing labor [35].

Some authors have argued for specialized formulas to be developed for specific fetal weight ranges. This applies to the formula by Hart et al. which was specifically developed for fetuses ≥ 4000 g. Using specialized formulas of this type appears at first to be a promising and plausible approach. For example, direct comparison between the Hart formula and the Hadlock formula for children with a BW ≥ 4000 g initially shows encouraging results. However, when one looks more closely at the “behavior” of the Hart formula, one realizes that the favorable sensitivity and specificity for neonates with a BW ≥ 4000 g is achieved by the way in which the Hart formula sets the estimation weights within a very narrow range around 4200 g (Fig. 1). On one hand, this leads to overestimation of fetal weight in neonates with a BW < 4000 g, and on the other hand it fails to recognize high-risk fetuses with a BW ≥ 4500 g or even ≥ 5000 g. In the present authors’ view, this characteristic of the Hart formula represents a serious disadvantage and may lead to unnecessary worry for mothers and obstetricians. Using the Hart formula will inevitably lead to more cesarean sections and labor inductions. This is not altered by the recommended AC cut-off value of 351 mm. Similarly, including maternal weight in the Hart formula has not been able to improve fetal weight measurement in obese women.

To date, obstetricians still do not have a viable tool for detecting macrosomic fetuses with satisfactory certainty. This is also reflected in the current ACOG *Practice Bulletin,* which states that “an accurate diagnosis of macrosomia can only be made by weighing the newborn after delivery” [35].

In our view, it is indispensable for every obstetrician to be aware of the weaknesses of the estimation formulas in use. Expectant parents also need to be informed about the limitations of fetal weight estimations, especially in cases of suspected macrosomia. All too easily, today’s highly sophisticated ultrasound equipment may lead to the misjudgment that we are able to estimate fetal weight better than is actually the case.

## Conclusion

For the important question of whether or not a fetus is macrosomic, Hart’s formula does not provide any additional benefit in comparison with the established Hadlock formula. It does not even offer any improvement in the group of obese mothers. In fetuses with a BW < 4000 g, the formula leads to weight estimates that are consistently too high. In addition, the Hart formula fails to detect fetuses with a BW ≥ 4500 g or even ≥ 5000 g. Uncritical implementation of the Hart formula may, therefore, lead to an increased rate of labor induction and CDs. On the basis of this analysis, it is arguable that the Hart formula has no place in screening for macrosomic fetuses in clinical practice.

## References

[1] Spellacy WN, Miller S, Winegar A, Peterson PQ (1985). Macrosomia—maternal characteristics and infant complications. Obstet Gynecol.

[2] Gudmundsson S, Henningsson A-C, Lindqvist P (2005). Correlation of birth injury with maternal height and birthweight. BJOG.

[3] Jolly MC, Sebire NJ, Harris JP, Regan L, Robinson S (2003). Risk factors for macrosomia and its clinical consequences: a study of 350,311 pregnancies. Eur J Obstet Gynecol Reprod Biol.

[4] Ecker JL, Greenberg JA, Norwitz ER, Nadel AS, Repke JT (1997). Birth weight as a predictor of brachial plexus injury. Obstet Gynecol.

[5] Gilbert WM, Nesbitt TS, Danielsen B (1999). Associated factors in 1611 cases of brachial plexus injury. Obstet Gynecol.

[6] Peleg D, Hasnin J, Shalev E (1997). Fractured clavicle and Erb’s palsy unrelated to birth trauma. Am J Obstet Gynecol.

[7] Zhang X, Decker A, Platt RW, Kramer MS (2008). How big is too big? The perinatal consequences of fetal macrosomia. Am J Obstet Gynecol.

[8] Weeks JW, Pitman T, Spinnato JA (1995). Fetal macrosomia: does antenatal prediction affect delivery route and birth outcome?. Am J Obstet Gynecol.

[9] Campbell S (2014). Fetal macrosomia: a problem in need of a policy. Ultrasound Obstet Gynecol.

[10] Coomarasamy A, Connock M, Thornton J, Khan KS (2005). Accuracy of ultrasound biometry in the prediction of macrosomia: a systematic quantitative review. BJOG.

[11] Boulet SL, Alexander GR, Salihu HM, Pass M (2003). Macrosomic births in the United States: determinants, outcomes, and proposed grades of risk. Am J Obstet Gynecol.

[12] Chauhan SP, Grobman WA, Gherman RA, Chauhan VB, Chang G, Magann EF (2005). Suspicion and treatment of the macrosomic fetus: a review. Am J Obstet Gynecol.

[13] Melamed N, Yogev Y, Meizner I, Mashiach R, Bardin R, Ben-Haroush A (2009). Sonographic fetal weight estimation: which model should be used?. J Ultrasound Med.

[14] Hadlock FP, Harrist RB, Sharman RS, Deter RL, Park SK (1985). Estimation of fetal weight with the use of head, body, and femur measurements—a prospective study. Am J Obstet Gynecol.

[15] Hart NC, Hilbert A, Meurer B, Schrauder M, Schmid M, Siemer J (2010). Macrosomia: a new formula for optimized fetal weight estimation. Ultrasound Obstet Gynecol.

[16] Bamberg C, Hinkson L, Henrich W (2013). Prenatal detection and consequences of fetal macrosomia. Fetal Diagn Ther.

[17] Hoopmann M, Abele H, Wagner N, Wallwiener D, Kagan KO (2010). Performance of 36 different weight estimation formulae in fetuses with macrosomia. Fetal Diagn Ther.

[18] Salomon LJ, Alfirevic Z, Berghella V, Bilardo C, Hernandez-Andrade E, Johnsen SL (2011). Practice guidelines for performance of the routine mid-trimester fetal ultrasound scan. Ultrasound Obstet Gynecol.

[19] R Development Core Team (2011) R: a language and environment for statistical computing. R Foundation for Statistical Computing Vienna. http://www.R-project.org/. Accessed 17 Apr 2018

[20] Stirnemann J, Villar J, Salomon LJ, Ohuma E, Ruyan P, Altman DG (2017). International estimated fetal weight standards of the INTERGROWTH-21st Project. Ultrasound Obstet Gynecol.

[21] Nahum GG, Stanislaw H, Huffaker BJ (1999). Accurate prediction of term birth weight from prospectively measurable maternal characteristics. J Reprod Med.

[22] Aviram A, Yogev Y, Ashwal E, Hiersch L, Hadar E, Gabbay-Benziv R (2017). Prediction of large for gestational age by various sonographic fetal weight estimation formulas—which should we use?. J Perinatol.

[23] Shepard MJ, Richards VA, Berkowitz RL, Warsof SL, Hobbins JC (1982). An evaluation of two equations for predicting fetal weight by ultrasound. Am J Obstet Gynecol.

[24] Merz E, Lieser H, Schicketanz KH, Härle J (1988). Intrauterine fetal weight assessment using ultrasound. A comparison of several weight assessment methods and development of a new formula for the determination of fetal weight. Ultraschall Med.

[25] Balsyte D, Schäffer L, Burkhardt T, Wisser J, Kurmanavicius J (2009). Sonographic prediction of macrosomia cannot be improved by combination with pregnancy-specific characteristics. Ultrasound Obstet Gynecol.

[26] Sadeh-Mestechkin D, Walfisch A, Shachar R, Shoham-Vardi I, Vardi H, Hallak M (2008). Suspected macrosomia? Better not tell. Arch Gynecol Obstet.

[27] Levine AB, Lockwood CJ, Brown B, Lapinski R, Berkowitz RL (1992). Sonographic diagnosis of the large for gestational age fetus at term: does it make a difference?. Obstet Gynecol.

[28] Parry S, Severs CP, Sehdev HM, Macones GA, White LM, Morgan MA (2000). Ultrasonographic prediction of fetal macrosomia. Association with cesarean delivery. J Reprod Med.

[29] Blackwell SC, Refuerzo J, Chadha R, Carreno CA (2009). Overestimation of fetal weight by ultrasound: does it influence the likelihood of cesarean delivery for labor arrest?. Am J Obstet Gynecol.

[30] Little SE, Edlow AG, Thomas AM, Smith NA (2012). Estimated fetal weight by ultrasound: a modifiable risk factor for cesarean delivery?. Am J Obstet Gynecol.

[31] Melamed N, Yogev Y, Meizner I, Mashiach R, Ben-Haroush A (2010). Sonographic prediction of fetal macrosomia: the consequences of false diagnosis. J Ultrasound Med.

[32] Henriksen T (2008). The macrosomic fetus: a challenge in current obstetrics. Acta Obstet Gynecol Scand.

[33] Sanchez-Ramos L, Bernstein S, Kaunitz AM (2002). Expectant management versus labor induction for suspected fetal macrosomia: a systematic review. Obstet Gynecol.

[34] Gonen R, Bader D, Ajami M (2000). Effects of a policy of elective cesarean delivery in cases of suspected fetal macrosomia on the incidence of brachial plexus injury and the rate of cesarean delivery. Am J Obstet Gynecol.

[35] American College of Obstetricians and Gynecologists’ Committee on Practice Bulletins—Obstetrics (2016). Practice bulletin no. 173: fetal macrosomia. Obstet Gynecol.

